# Metabolic Dysfunction‐Associated Steatohepatitis and the Progression of Cognitive Decline in Older Thai People With HIV

**DOI:** 10.1002/jia2.70215

**Published:** 2026-10-08

**Authors:** Katekarn Porkaew, Akarin Hiransuthikul, Win Min Han, Tanakorn Apornpong, Sasiwimol Ubolyam, Stephen Kerr, Anchalee Avihingsanon, HIV‐NAT 006 and 207 study team

**Affiliations:** ^1^ Department of Preventive and Social Medicine Faculty of Medicine Chulalongkorn University Bangkok Thailand; ^2^ HIV‐NAT Thai Red Cross AIDS and Infectious Diseases Research Centre Bangkok Thailand; ^3^ Kirby Institute UNSW Sydney Sydney New South Wales Australia; ^4^ Center of Excellence in Tuberculosis Department of Medicine Faculty of Medicine Chulalongkorn University Bangkok Thailand; ^5^ Biostatistics Excellence Centre Faculty of Medicine Chulalongkorn University Bangkok Thailand

**Keywords:** ageing, cognition, human immunodeficiency virus, metabolic dysfunction‐associated steatohepatitis, metabolic dysfunction‐associated steatotic liver disease, Thailand

## Abstract

**Introduction:**

Metabolic dysfunction‐associated steatohepatitis (MASH) is more common in people with HIV and associated with systemic inflammation and extrahepatic complications, including cognitive impairment. While cross‐sectional studies suggest an association between MASH and worse cognitive outcomes, its impact on longitudinal cognitive trajectories in people with HIV remains unclear.

**Methods:**

This prospective study included people with HIV aged≥50 years from HIV‐NAT, Bangkok, Thailand, between 2015 and 2024. MASLD and MASH were identified at baseline using the FibroScan‐AST (FAST) score. Cognitive function was assessed using the Thai‐validated Montreal Cognitive Assessment (MoCA) at baseline and a follow‐up visit. A multivariate linear mixed model was used to determine the difference in changes in MoCA scores between participants with and without baseline MASH.

**Results:**

Among 291 participants (60.5% male; median age 54.4 years), 60 (20.6%) met criteria for MASH at baseline. Over a median follow‐up of 5.9 years, baseline MASLD was not associated with cognitive decline, whereas participants with baseline MASH showed a significantly greater cognitive decline between ages 50 and 80 years, after adjusting for age, sex, body mass index, education level, employment status, smoking, alcohol consumption, diabetes, hypertension, duration of HIV acquisition and exposure to efavirenz. Participants with MASH showed a mean MoCA decline of –4.79 (95% CI –7.49 to –2.09), compared with –0.94 (95% CI –2.20 to –0.32) among those without MASH (*p*‐interaction = 0.007).

**Conclusions:**

MASH is a significant predictor of steeper cognitive decline in older people with HIV, independent of traditional metabolic and HIV‐related risk factors. These findings suggest that early identification and management of MASH may help mitigate cognitive decline in ageing HIV care.

## 1 Introduction

Metabolic dysfunction‐associated steatotic liver disease (MASLD), formerly known as non‐alcoholic fatty liver disease (NAFLD) or metabolic dysfunction‐associated fatty liver disease (MAFLD), has become increasingly prevalent worldwide, affecting approximately 25%–40% of the global adult population, with prevalence varying by country [1, 2]. Within the MASLD spectrum, metabolic dysfunction‐associated steatohepatitis (MASH) represents the progressive inflammatory phenotype and is associated with more severe hepatic complications such as liver fibrosis, cirrhosis and hepatocellular carcinoma [2, 3, 4]. MALSD prevalence among people with HIV is around 35%–64%, depending on the diagnostic criteria used, and appears comparable to that of the general population [5, 6, 7, 8]. In contrast, the burden of MASH is disproportionally higher among people with HIV than normal population, with the prevalence of up to 42% [8, 9]. This elevated risk is likely driven by a combination of HIV‐related immune activation and systemic inflammation, metabolic effects of antiretroviral therapies (ARTs), gut dysbiosis and bacterial translocation, and traditional cardiometabolic risk factors [10, 11, 12]. Beyond hepatic manifestations, MASLD and MASH are increasingly recognized as systemic conditions associated with a range of extrahepatic complications, including cognitive impairment [13, 14, 15]. In particular, MASH may be more strongly associated with cognitive impairment because it represents the progressive inflammatory phenotype of MASLD.

Advances in ART have substantially improved life expectancy in people with HIV, leading to increased prevalence of chronic and degenerative diseases [16, 17]. As a result, the prevalence of cognitive impairment among people with HIV has increased as these cohorts age [18]. Though the exact mechanisms remain unclear, proposed contributors include HIV‐related persistent immune dysregulation, heightened cardiovascular risk factors, and lifestyle‐related factors such as tobacco use and substance abuse [6, 19, 20, 21]. In the general population, several studies have reported an association between MASLD and poorer cognitive performance [22, 23, 24, 25, 26], raising concern that liver‐related metabolic and inflammatory processes may contribute to cognitive decline [27]. However, conflicting data have been reported. Findings from the ELSA‐Brasil cohort, a large multicentre study conducted in Brazil, showed no independent association between MASLD and cognitive decline after adjusting for cardiometabolic risk factors [28].

Despite these observations, the association between MASLD and MASH and cognitive impairment in people with HIV still remains unclear. Existing studies in people with HIV are limited and are mostly cross‐sectional, with some suggesting an association between liver steatosis and cognitive impairment in people with HIV [29, 30]. However, longitudinal evidence examining cognitive trajectories in relation to MASLD and MASH remains limited. Longitudinal assessment is critical to determine whether MASLD/MASH contributes to the progression of cognitive decline over time. Therefore, this study aimed to investigate the longitudinal association between baseline MASLD and MASH and cognitive changes among older people with HIV, with the goal of improving understanding of how liver metabolic dysfunction may influence cognitive trajectories and guide future prevention and management strategies.

## 2 Methods

### 2.1 Study Design and Study Population

This study used data from HIV‐NAT 207 (Aging Study), an ageing sub‐cohort of the ongoing HIV‐NAT 006 cohort, a prospective observational cohort of adults with HIV established in 1996 (NCT00411983). HIV‐NAT 207 enrolled people with HIV aged ≥50 years between 2015 and 2017 to investigate age‐related comorbidities.

For this analysis, baseline was defined as the HIV‐NAT 207 enrolment visit (2015–2017), during which participants underwent transient elastography (TE) to assess liver stiffness measurement (LSM) and controlled attenuation parameter (CAP), as well as cognitive assessment using the Thai‐validated Montreal Cognitive Assessment (MoCA). The follow‐up visit for the second MoCA took place between 2021 and 2024. People with HIV who completed TE at baseline and MoCA assessments at both baseline and follow‐up visits were included in this analysis.

All participants provided written informed consent prior to their participation. The study protocol was approved by the Institutional Review Board of the Faculty of Medicine, Chulalongkorn University, Bangkok, Thailand (IRB No. 442/58).

### 2.2 Definitions and Measurements of Study Variables

MASLD was diagnosed using TE, which included CAP and LSM, performed by two trained and experienced technicians using FibroScan and a standard M probe for participants with a normal body mass index (BMI). The diagnosis was based on these imaging findings in combination with cardiometabolic criteria, including overweight or obesity (BMI ≥23 kg/m^2^ for Asians), dysglycaemia or type 2 diabetes, elevated triglycerides (≥150 mg/dL) or on lipid‐lowering treatment, low HDL‐cholesterol (≤39 mg/dL in men, ≤50 mg/dL in women) and high blood pressure (≥130/85 mmHg) or on treatment for hypertension [31]. The diagnostic approach to the disease spectrum of steatotic liver disease (SLD) depends on the primary clinical objective: CAP ≥248 dB/m is used to detect steatosis, LSM ≥12 kPa for F3 severe fibrosis and the FibroScan‐AST (FAST) score ≥0.67 to identify individuals at risk of developing MASH, a more advanced stage in the spectrum of MASLD [31]. The FAST score, combining CAP and LSM from TE and AST levels, has been utilized in some recent studies involving people with HIV [9, 30, 32, 33, 34, 35]. FAST score was calculated by using the following formula [36]: $\mathrm{FAST}\;=\frac{e^{-1.65+1.07\times \ln\left(\mathrm{LS}\right)+2.66*10^{-8}\times \mathrm{CA}P^{3}-63.3\times \mathrm{AS}T^{-1}}}{1+e^{-1.65+1.07\times \ln\left(\mathrm{LS}\right)+2.66*10^{-8}\times \mathrm{CA}P^{3}-63.3\times \mathrm{AS}T^{-1}}},$ where LS means liver stiffness measurement, CAP means controlled attenuation parameter and AST means aspartate aminotransferase level.

The FAST score is better at detecting patients at risk of MASH, which is useful in clinical decision‐making for further evaluation and management. The score has been validated against liver biopsy in multiple international cohorts, showing good diagnostic performance (area under the receiver operating characteristic curve [AUROC] 0.74–0.95), using a dual cut‐off with FAST ≥0.67 to rule in and ≤0.35 to rule out non‐alcoholic steatohepatitis (NASH, the previous term of MASH), elevated NAFLD activity score (NAS ≥4) and advanced fibrosis (F2 or higher) [36].

The Thai‐validated version of the MoCA adjusted the cut‐off score from 26 to 25 and added an additional point to participants with ≤6 years of education instead of ≤12, as in the original MoCA [37]. Cognitive impairment was defined as a score of less than 25.

The primary exposures were baseline MASH and MASLD, both analysed as categorical variables (presence or absence). The primary outcome was cognitive performance measured using the MoCA, which was analysed as a continuous variable in the multivariate linear mixed models. Potential confounders included age, sex, body mass index, education level, employment status, smoking, alcohol consumption, diabetes, hypertension, duration of HIV acquisition and exposure to efavirenz (EFV).

### 2.3 Statistical Analysis

Demographic characteristics at baseline were summarized as frequencies with percentages for categorical data and medians with interquartile ranges (IQR) for continuous data. Multivariate linear mixed models were used to examine the differences in changes in MoCA scores between baseline and follow‐up according to baseline MASH and MASLD status. MoCA score was used as a continuous outcome variable. The models included participant‐specific random intercepts to account for within‐subject correlation between repeated MoCA measurements. Fixed effects included baseline MASH or MASLD status, age, and potential confounders. An interaction term between baseline MASH or MASLD status and age was used to assess whether model‐estimated changes in MoCA scores across the age range differed by baseline MASH or MASLD status. Given that cognitive function was assessed at only two time points, the mixed models were used to estimate differences in cognitive changes between baseline and follow‐up rather than detailed longitudinal trajectories.

Two models were constructed, and covariates were selected a priori based on previously reported data to be potential confounders of the relationships between MASH or MASLD and cognitive performance. Model I, adjusted for age, sex, body mass index, education level, employment status, smoking, alcohol consumption, diabetes and hypertension; and Model II, adjusted for the same variables as Model I with the addition of HIV‐related factors, including duration of HIV acquisition and exposure to EFV. The analytic approach was adapted from a recent longitudinal study evaluating blood‐based Alzheimer's disease biomarkers and cognitive trajectories among older people with HIV [38]. Statistical significance was set at *p*‐value <0.05. All analyses were conducted in Stata/SE 17.0 (StataCorp LP, College Station, TX, USA).

## 3 Results

Among 291 participants, 60.5% were male, with a median age of 54.4 years (IQR: 51.8–59.1). At baseline, 20.6% met criteria for MASH. The median duration of HIV acquisition was 18.2 years (IQR: 15.0–20.9), and 97.6% had plasma HIV RNA ≤50 copies/mL. The median CD4 cell count at baseline was 618.5 (IQR: 493.0–803.0). 94.5% of participants were receiving ART regimens containing nucleoside reverse transcriptase inhibitors (NRTIs); 61.5% were receiving non‐nucleoside reverse transcriptase inhibitors (NNRTIs); 43.6% were receiving protease inhibitors (PIs); and 5.2% were receiving integrase strand transfer inhibitors (INSTIs). 58.8% of participants were exposed to EFV. A small proportion of participants had hepatitis B virus (10.3%) or hepatitis C virus (8.9%), all of whom had received treatment. A total of 26 participants (9%) reported alcohol use, but none met the criteria for excessive alcohol consumption (>20 g/day for women or >30 g/day for men). Baseline characteristics, including MoCA scores, were comparable between participants with and without MASH (Table 1).

**TABLE 1:** Baseline characteristics of 291 participants included in the analysis.

Characteristics	Total (*n* = 291) *n* (%)	No MASH (*n* = 231) *n* (%)	MASH (*n* = 60) *n* (%)
Age	54.4 (51.8, 59.1)	54.4 (52.0, 59.0)	53.7 (51.4, 60.0)
Sex			
Male	176 (60.5%)	132 (57.1%)	44 (73.3%)
Female	115 (39.5%)	99 (42.9%)	16 (26.7%)
BMI (kg/m^2^)	23.0 (20.7, 25.0)	22.8 (20.5, 24.7)	23.5 (21.4, 26.1)
Education			
Education ≤6 years	74 (25.4%)	60 (26.0%)	14 (23.3%)
Education >6 years	217 (74.6%)	171 (74.0%)	46 (76.7%)
Employment			
Unemployed	55 (18.9%)	45 (19.5%)	10 (16.7%)
Employed	236 (81.1%)	186 (80.5%)	50 (83.3%)
Income (per month)^a^			
No income	29 (10.0%)	24 (10.4%)	5 (8.3%)
<10,000 THB (319 USD)	87 (29.9%)	67 (29.0%)	20 (33.3%)
10,000−19,999 THB (320−636 USD)	88 (30.2%)	74 (32.0%)	14 (23.3%)
≥20,000 THB (367 USD)	87 (29.9%)	66 (28.6%)	21 (35.0%)
Smoking status			
Never	194 (66.9%)	160 (69.6%)	34 (56.7%)
Ex‐smoker	56 (19.3%)	42 (18.3%)	14 (23.3%)
Currently smoking	40 (13.8%)	28 (12.2%)	12 (20.0%)
Alcohol consumption			
Never	233 (80.3%)	185 (80.4%)	48 (80.0%)
Ex‐drinker	31 (10.7%)	24 (10.4%)	7 (11.7%)
Currently drinking	26 (9.0%)	21 (9.1%)	5 (8.3%)
HBV coinfection	30 (10.3%)	24 (10.4%)	6 (10.0%)
HCV coinfection	26 (8.9%)	16 (6.9%)	10 (16.7%)
Diabetes mellitus	43 (14.8%)	33 (14.3%)	10 (16.7%)
Hypertension	117 (40.2%)	91 (39.4%)	26 (43.3%)
LDL cholesterol	124.6 (99.8, 147.6)	123.6 (99.8, 147.2)	125.3 (99.1, 149.6)
CAP (dB/m)	230.0 (190.0, 271.0)	222.0 (185.0, 257.0)	288.0 (219.0, 329.5)
LSM (kPa)	5.5 (4.4, 6.8)	5.1 (4.2, 6.1)	7.1 (5.8, 9.6)
SLD (CAP ≥248 dB/m)	110 (37.8%)	70 (30.3%)	40 (66.7%)
Advanced liver fibrosis (LSM ≥9.5 kPa)	27 (9.3%)	11 (4.8%)	16 (26.7%)
ALT (IU/mL)	28.0 (21.0, 40.0)	25.0 (20.0, 33.0)	49.5 (35.5, 83.0)
AST (IU/mL)	25.0 (21.0, 31.0)	24.0 (20.0, 28.0)	39.0 (30.0, 61.0)
FAST score	0.3 (0.2, 0.6)	0.2 (0.1, 0.4)	1.1 (0.8, 1.8)
Duration of HIV acquisition (years)	18.2 (15.0, 20.9)	18.8 (15.4, 20.9)	16.9 (13.5, 20.6)
Duration of ART	16.1 (12.4, 18.9)	16.3 (12.8, 19.2)	15.7 (11.3, 16.9)
Nadir CD4 (cells/mm^3^)	182.0 (92.0, 259.0)	182.0 (92.0, 256.0)	178.5 (80.5, 268.0)
Absolute CD4 count (cells/mm^3^)	618.5 (493.0, 803.0)	603.0 (493.0, 790.0)	695.0 (490.0, 861.0)
HIV‐1 RNA <50 copies/mL	282 (97.6%)	224 (97.4%)	58 (98.3%)
Types of ART			
NRTI	275 (94.5%)	217 (93.9%)	58 (96.7%)
NNRTI	179 (61.5%)	134 (58.0%)	45 (75.0%)
PI	127 (43.6%)	111 (48.1%)	16 (26.7%)
INSTI	15 (5.2%)	14 (6.1%)	1 (1.7%)
Ever exposed to EFV	171 (58.8%)	137 (59.3%)	34 (56.7%)
Total MoCA score	24.0 (21.0, 26.0)	24.0 (21.0, 26.0)	24.0 (21.5, 26.0)
Visuospatial/executive function	4.0 (3.0, 5.0)	4.0 (3.0, 5.0)	4.0 (3.0, 5.0)
Naming	3.0 (3.0, 3.0)	3.0 (3.0, 3.0)	3.0 (3.0, 3.0)
Attention	6.0 (5.0, 6.0)	6.0 (4.0, 6.0)	5.0 (5.0, 6.0)
Language	2.0 (1.0, 2.0)	2.0 (1.0, 2.0)	2.0 (1.0, 2.0)
Abstraction	2.0 (1.0, 2.0)	2.0 (1.0, 2.0)	2.0 (1.0, 2.0)
Delayed recall	3.0 (1.0, 4.0)	3.0 (1.0, 4.0)	3.0 (1.0, 4.0)
Orientation	6.0 (6.0, 6.0)	6.0 (6.0, 6.0)	6.0 (6.0, 6.0)
MoCA score <25	174 (59.8%)	139 (60.2%)	35 (58.3%)

*Note*: Categorical data are presented as frequency (percentage) and continuous data are presented as median (interquartile range).

Abbreviations: ALT, alanine aminotransferase; ART, antiretroviral therapy; AST, aspartate aminotransferase; BMI, body mass index; CAP, controlled attenuation parameter; EFV, efavirenz; FAST, FibroScan‐AST; HBV, hepatitis B virus; HCV, hepatitis C virus; INSTI, integrase strand transfer inhibitor; LDL cholesterol, low‐density lipoprotein cholesterol; LSM, liver stiffness measurement; MASH, metabolic dysfunction‐associated steatohepatitis; MoCA, Montreal Cognitive Assessment; NNRTI, non‐nucleoside reverse transcriptase inhibitor; PI, protease inhibitor; SLD, steatotic liver disease; THB, Thai baht; USD, United States dollar.

^a^ Currency was converted from THB to USD using an exchange rate of 1 THB = 0.032 USD (rounded to whole numbers).

Over a median interval of 5.9 years (IQR: 5.6–6.8) between the two MoCA, 150 (51.6%) participants had a decline in MoCA scores (53.3% among those with MASH vs. 51.1% among those without MASH, *p* = 0.756). At follow‐up, the median MoCA score was 22 (range: 11–28) in participants with MASH and 23 (range: 12–30) in those without MASH. MoCA scores <18 were observed in five participants (8.3%) with MASH and 23 participants (10.0%) without MASH. In both models, baseline MASH was associated with significantly greater cognitive decline between ages 50 and 80 years. In Model I, participants with MASH showed a mean MoCA decline of –5.47 (95% CI –8.14 to –2.80), compared with –1.46 (95% CI –2.65 to –0.28) among those without MASH (*p*‐interaction = 0.005). Similar findings were observed in Model II (–5.35 [95% CI –8.03 to –2.66] vs. –1.27 [95% CI –2.48 to –0.05]) (Figure 1). Baseline MASH was also significantly associated with a greater decline in the language subdomain in Model I (coefficient –0.034 [95% CI –0.068 to –0.001]), visuospatial/executive function subdomain in Model II (coefficient –0.045 [95% CI –0.088 to –0.002]) and orientation subdomain in both models (Model I: coefficient –0.026 [95% CI –0.041 to –0.011]; Model II: coefficient –0.026 [95% CI –0.042 to –0.011]) (Table 2). Excluding participants living with hepatitis B virus (HBV) or hepatitis C virus (HCV) yielded consistent findings.

**FIGURE 1:**
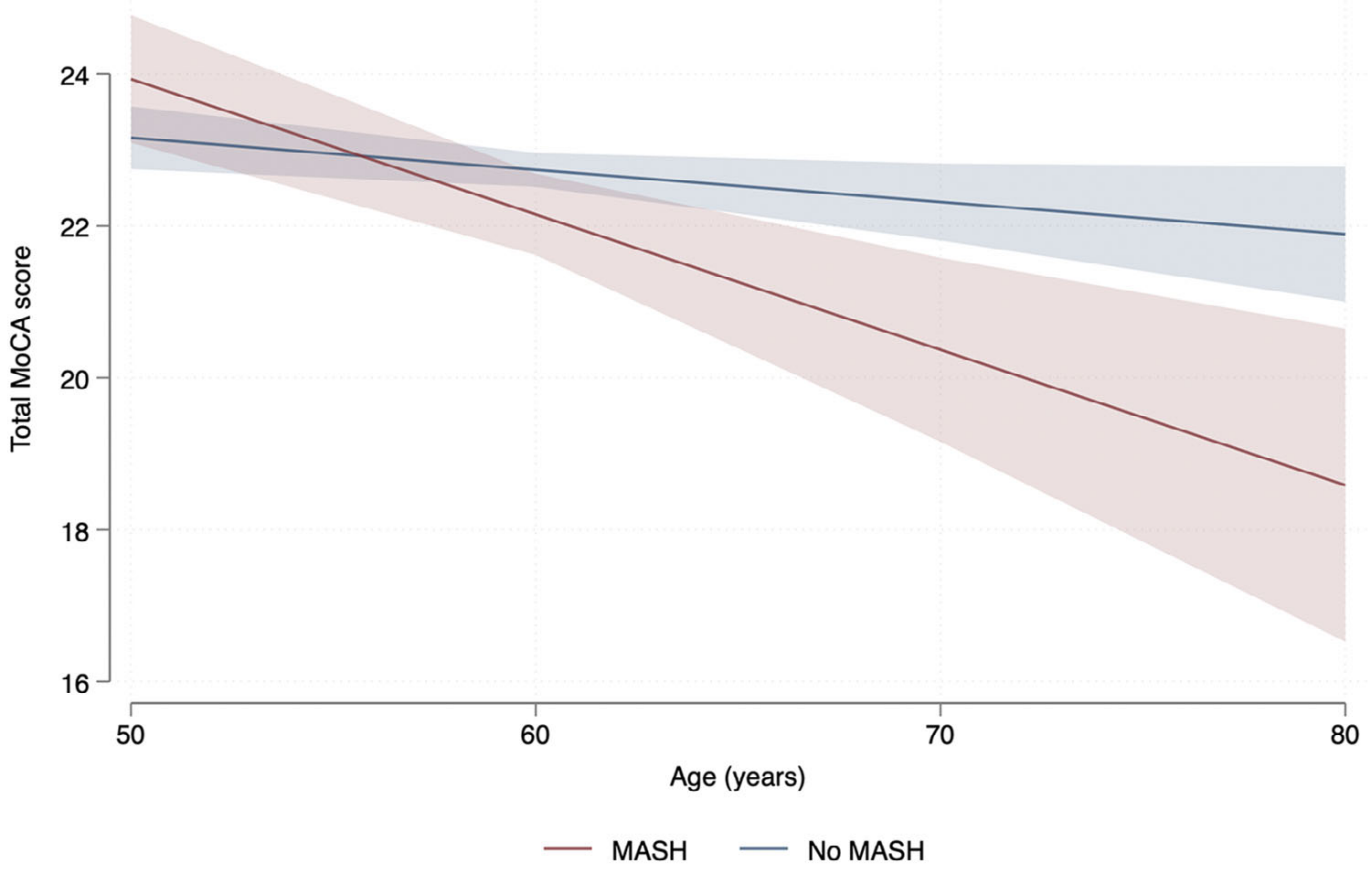
Modelled‐estimated changes in MoCA scores with 95% confidence interval from age 50 to 80 years in people with HIV with and without MASH at baseline. Adjusted for sex, body mass index, education level, employment status, smoking, alcohol consumption, diabetes, hypertension, HIV duration, exposure to efavirenz and baseline MoCA score. MASH, metabolic dysfunction‐associated steatohepatitis; MoCA, Montreal Cognitive Assessment.

**TABLE 2:** Adjusted coefficients with 95% confidence intervals for longitudinal changes in MoCA total score and cognitive subdomains according to baseline MASLD/MASH status.

MoCA score domain	Model I^a^			Model II^b^		
	Coefficient	95% CI	*p*	Coefficient	95% CI	*p*
**MASH**						
Total MoCA score	−0.133	(–0.227, –0.040)	0.005	−0.136	(–0.230, –0.042)	0.005
Visuospatial/executive function	−0.040	(–0.083, 0.003)	0.070	−0.045	(–0.088, –0.002)	0.038
Naming	0.001	(–0.016, 0.018)	0.913	0.002	(–0.016, 0.019)	0.848
Attention	−0.011	(–0.051, 0.028)	0.571	−0.012	(–0.052, 0.027)	0.541
Language	−0.034	(–0.068, –0.001)	0.047	−0.032	(–0.066, 0.002)	0.067
Abstraction	−0.016	(–0.044, 0.011)	0.247	−0.015	(–0.043, 0.012)	0.269
Delayed recall	−0.009	(–0.068, 0.049)	0.751	−0.007	(–0.065, 0.052)	0.827
Orientation	−0.026	(–0.041, –0.011)	0.001	−0.026	(–0.042, –0.011)	0.001
**MASLD**						
Total MoCA score	0.002	(–0.071, 0.075)	0.956	−0.008	(–0.082, 0.065)	0.829
Visuospatial/executive function	−0.005	(–0.039, 0.029)	0.772	−0.012	(–0.046, 0.021)	0.470
Naming	0.003	(–0.011, 0.017)	0.654	0.003	(–0.011, 0.017)	0.654
Attention	0.009	(–0.022, 0.040)	0.574	0.009	(–0.022, 0.041)	0.557
Language	−0.007	(–0.033, 0.020)	0.624	−0.008	(–0.035, 0.019)	0.551
Abstraction	−0.016	(–0.038, 0.005)	0.139	−0.019	(–0.040, 0.003)	0.089
Delayed recall	0.022	(–0.024, 0.068)	0.346	0.024	(–0.022, 0.070)	0.305
Orientation	0.005	(–0.007, 0.018)	0.380	0.005	(–0.007, 0.018)	0.390

Abbreviations: MASH, metabolic dysfunction‐associated steatohepatitis; MASLD, metabolic dysfunction‐associated steatotic liver disease; MoCA, Montreal Cognitive Assessment.

^a^ Model I was adjusted for age, sex, body mass index, education level, employment status, smoking, alcohol consumption, diabetes and hypertension.

^b^ Model II was adjusted for the same covariates as Model I *plus* duration of HIV acquisition, exposure to efavirenz.

Baseline MASLD was not significantly associated with differences in longitudinal changes of total MoCA scores (Model I: coefficient 0.002 [95% CI –0.071 to 0.075]; Model II: coefficient –0.008 [95% CI –0.082 to 0.065]) or individual MoCA subdomains in either model (Figure 2 and Table 2).

**FIGURE 2:**
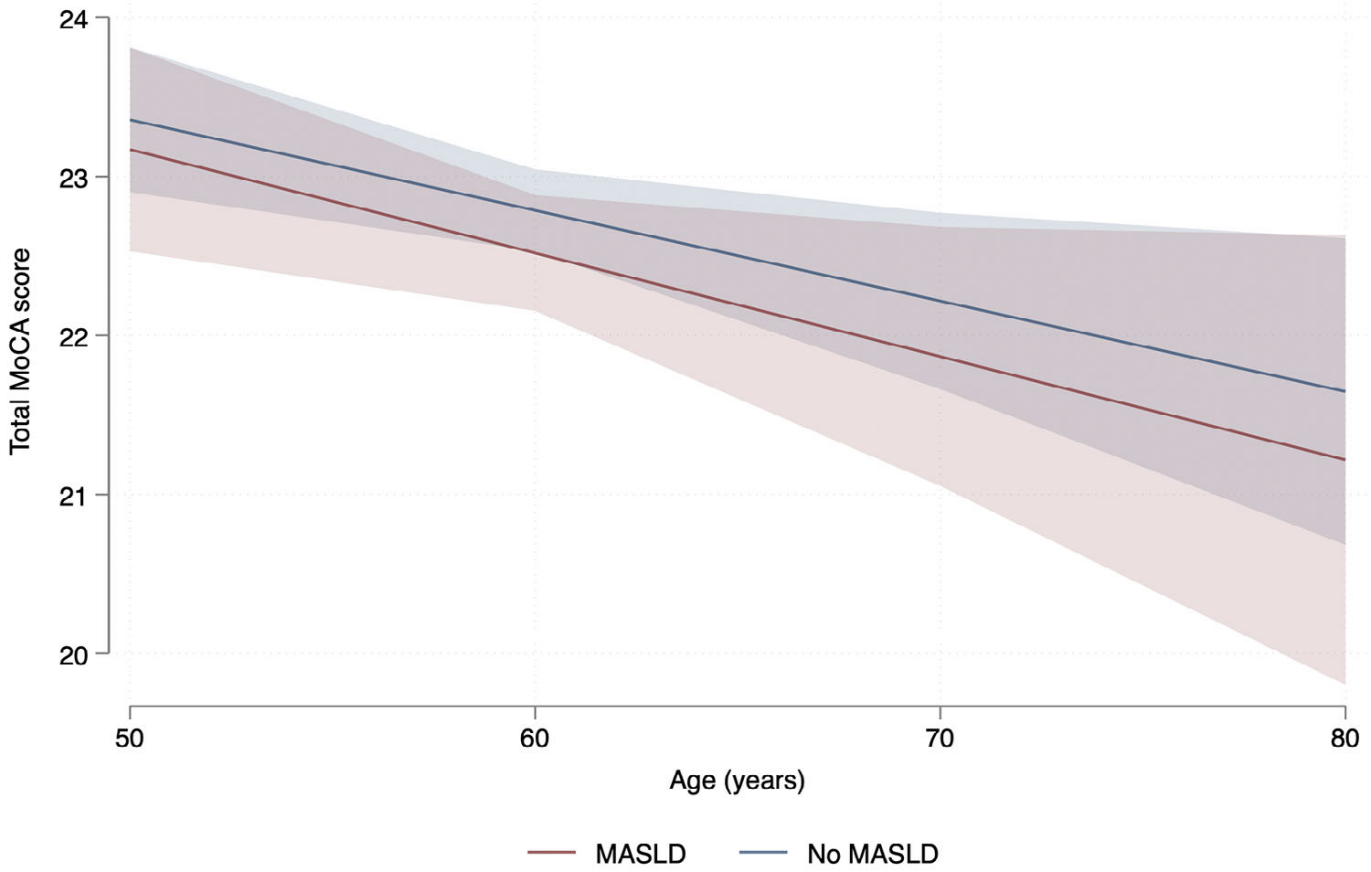
Modelled‐estimated changes in MoCA scores with 95% confidence interval from age 50 to 80 years in people with HIV with and without MASLD at baseline. Adjusted for sex, body mass index, education level, employment status, smoking, alcohol consumption, diabetes, hypertension, HIV duration, exposure to efavirenz and baseline MoCA score. MASLD, metabolic dysfunction‐associated steatotic liver disease; MoCA, Montreal Cognitive Assessment.

## 4 Discussion

In this prospective cohort of older people with HIV, we found that baseline MASH was associated with significantly greater cognitive decline over time compared to those without MASH. In contrast, MASLD was not associated with cognitive decline. Importantly, the magnitude of decline associated with MASH appears clinically meaningful, with adjusted models estimating an approximately 5–6 point decline in MoCA score across the modelled age range of 50–80 years, compared with less than a 1–2 point decline among those without MASH. Given that the MoCA is scored on a 30‐point scale, this degree of decline suggests substantially greater worsening in global cognitive function among participants with MASH.

These findings are consistent with observations from the ELSA‐Brasil cohort, which reported no independent association between MASLD and cognitive decline in middle‐aged adults [28]. Together, these findings suggest that progressive inflammatory liver disease, rather than steatosis alone, may play a critical role in neurocognitive deterioration among ageing people with HIV. One possible explanation is that MASH represents the inflammatory stage of MASLD. Unlike steatosis alone, MASH is characterized by hepatocellular injury, inflammation and fibrogenesis, which may promote systemic inflammation and subsequent neurocognitive impairment.

Previous studies have reported associations between liver steatosis and cognitive impairment in both the general population and in people with HIV [22, 23, 24, 25, 26, 29, 30]; however, most were cross‐sectional. Our longitudinal study extends previous findings by demonstrating that MASH, defined using the non‐invasive FAST score, independently predicts future cognitive decline. In our cohort, participants with hepatitis B and/or C coinfection received treatment; therefore, viral hepatitis was unlikely to contribute significantly to liver‐related complications. Additionally, none of the participants who reported alcohol consumption met the criteria for excessive alcohol drinking, indicating a minimal risk of including individuals with alcohol‐related liver disease (ALD) or metabolic and alcohol‐related liver disease (MetALD).

Given that MASLD was not significantly associated with cognitive changes over time, whereas MASH showed a robust association, these findings suggested that liver inflammation and liver injury—rather than steatosis alone—may contribute to cognitive decline over time, especially in the context of ageing and chronic HIV acquisition. This interpretation is consistent with prior evidence suggesting that MASH‐related metabolic and inflammatory processes, including systemic inflammation, oxidative stress and endothelial dysfunction, may promote neurodegeneration and cerebrovascular injury [30].

MASH is characterized by hepatic steatosis, lobular inflammation and hepatocellular injury [31]. Hepatic steatosis and inflammation can promote systemic release of pro‐inflammatory cytokines, causing a systemic inflammatory response [21, 24, 26]. Such chronic low‐grade inflammation may impair blood−brain barrier integrity, activate microglia and cause subsequent neuroinflammation, all of which can lead to neuronal damage and cognitive deterioration [24]. Moreover, insulin resistance—a shared pathophysiological feature of both MAFLD/MASH and Alzheimer's disease—may further contribute to cognitive decline through impaired glucose metabolism and increased oxidative stress [26]. These pathways may be especially relevant in people with HIV, who experience persistent immune activation, chronic inflammation and metabolic dysregulation despite effective ART [20, 30]. Emerging evidence also highlights the role of the brain−gut−liver axis, a complex inter‐organ communication pathway which links the hepatic, intestinal and central nervous system together [27, 39, 40]. Dysregulation of this axis via gut microbiota dysbiosis, bacterial translocation and hepatic dysfunction can impact neurocognitive outcomes through immune, enteroendocrine and neuroendocrine signalling pathways [27, 39, 40].

Some limitations should be acknowledged. First, MASH was defined using a non‐invasive FAST score rather than histological confirmation by liver biopsy, which may have led to misclassification and residual confounding. In addition, transient elastography was performed only at baseline, limiting the assessment of their progression or regression during follow‐up, and we were unable to account for certain lifestyle‐related factors or other management strategies, which may influence both liver disease severity and cognitive outcomes. Second, cognitive function was assessed at only two time points, limiting detailed assessment of longitudinal cognitive change; therefore, the reported cognitive changes across ages 50−80 years represent model‐estimated trajectories rather than directly observed long‐term changes. Cognitive outcomes were also assessed using MoCA alone without comprehensive neuropsychological battery testing, limiting detailed evaluation of domain‐specific cognitive impairment. Finally, we were unable to examine pathophysiological processes, such as systemic inflammation, neuroinflammation, or liver fibrosis by using imaging or biomarker‐based measures. Further studies integrating imaging and biomarker assessment could provide a deeper understanding of the pathways underlying the relationship between MASH and cognitive outcomes.

## 5 Conclusions

In conclusion, baseline MASH was associated with steeper cognitive decline in older people with HIV, independent of traditional metabolic and HIV‐related factors. These findings emphasize the importance of recognizing and managing metabolic liver disease in ageing people with HIV populations, not only to prevent liver‐related outcomes but also to potentially reduce the risk of cognitive decline. Whether interventions targeting MASH can alter the course of cognitive decline remains unclear and requires further investigation. In addition, larger studies in people with HIV are needed to confirm these findings and further clarify the underlying mechanisms.

## Author Contributions

KP drafted the initial version of the manuscript. KP, AH and WMH conceptualized the study. AH performed the statistical analyses. AH, WMH, TA and SK contributed to data interpretation and supported the statistical analyses. TA contributed to data management. SU facilitated laboratory management. AA led the study. All authors reviewed and approved the final manuscript.

## Funding

This study was supported by the Higher Education Research Promotion and National Research University Project of Thailand, Office of the Higher Education Commission and Chulalongkorn University (Project code NRU59‐(014)‐ HR).

## Conflicts of Interest

AH received research grants from Gilead Sciences. AA has received research grants for HIV‐NAT from Gilead Sciences, ViiV/GSK, Roche, MSD, Janssen Research & Development; transportation costs to attend meetings/conferences from Gilead Sciences; and other non‐financial interests (unpaid) for working with: (1) Strategic and Technical Advisory group to WHO for HIV/hepatitis/STI; (2) Thai AIDS society committee; and (3) Thailand National ART, TB, HIV, Hepatitis programme committee. The rest of the authors declare no relevant conflicts of interest.

## Data Availability

The data that support the findings of this study are available on request from the corresponding author. The data are not publicly available due to privacy or ethical restrictions.
